# NGS panel enhance precise diagnosis of myeloid neoplasms under WHO-HAEM5 and International Consensus Classification: An observational study

**DOI:** 10.1097/MD.0000000000038556

**Published:** 2024-06-14

**Authors:** Xiangjun Ye, Zhikang Zheng, Yuwei Wu, Zhihui Zhang, Zhiping Xu, Yameng Liu, Lei Jiang, Jianguo Wu

**Affiliations:** a Department of Laboratory Medicine, the Fourth Affiliated Hospital of School of Medicine, and International School of Medicine, International Institutes of Medicine, Zhejiang University, Yiwu, China; b Social and Behavioral Sciences, University of Amsterdam, Netherlands; c Department of Hematology, the Fourth Affiliated Hospital of School of Medicine, and International School of Medicine, International Institutes of Medicine, Zhejiang University, Yiwu, China; d Department of Laboratory Medicine of Zhejiang Provincial People’s Hospital, Hangzhou, China; e Department of Laboratory Medicine, Hangzhou TCM Hospital Affiliated to Zhejiang Chinese Medical University, Hangzhou, Zhenjiang, China.

**Keywords:** 5th edition of WHO classification, gene mutation, International Consensus Classification, myeloid neoplasms, next-generation sequencing

## Abstract

This study aimed to assess hematological diseases next-generation sequencing (NGS) panel enhances the diagnosis and classification of myeloid neoplasms (MN) using the 5th edition of the WHO Classification of Hematolymphoid Tumors (WHO-HAEM5) and the International Consensus Classification (ICC) of Myeloid Tumors. A cohort of 112 patients diagnosed with MN according to the revised fourth edition of the WHO classification (WHO-HAEM4R) underwent testing with a 141-gene NGS panel for hematological diseases. Ancillary studies were also conducted, including bone marrow cytomorphology and routine cytogenetics. The cases were then reclassified according to WHO-HAEM5 and ICC to assess the practical impact of these 2 classifications. The mutation detection rates were 93% for acute myeloid leukemia (AML), 89% for myelodysplastic syndrome (MDS), 94% for myeloproliferative neoplasm (MPN), and 100% for myelodysplasia/myeloproliferative neoplasm (MDS/MPN) (WHO-HAEM4R). NGS provided subclassified information for 26 and 29 patients with WHO-HAEM5 and ICC, respectively. In MPN, NGS confirmed diagnoses in 16 cases by detecting *JAK2, MPL*, or *CALR* mutations, whereas 13 “triple-negative” MPN cases revealed at least 1 mutation. NGS panel testing for hematological diseases improves the diagnosis and classification of MN. When diagnosed with ICC, NGS produces more classification subtype information than WHO-HAEM5.

## 1. Introduction

Myeloid neoplasms (MN) are neoplastic diseases caused by the clonal proliferation of myeloid cells, which usually involve the bone marrow and affect blood cells, thus affecting the functions of other systems and even leading to short-term death. The diagnosis of MN is complicated, mainly relying on the World Health Organization (WHO) tumor classification.^[1,2]^ Next-generation sequencing (NGS), as a high-throughput technology, can sequence DNA or RNA quickly and cost-effectively, making it suitable for simultaneous detection of multiple genetic abnormalities in MN. The sequence variants/mutations it detects are widely used in MN diagnosis, prognosis, treatment decisions, and patient follow-up.^[3]^ Since the publication of the revised 4th edition of the WHO classification of hematolymphoid tumors (WHO-HAEM4R) in 2016, NGS mutation analysis has become common in clinical practice. The basis for MN classification continues to evolve as new pathological features are discovered and new therapies become available. Therefore, the World Health Organization released the fifth edition of the World Health Organization Classification of Hemolymph Neoplasms (WHO-HAEM5) on June 22, 2022.^[4]^ Shortly thereafter, the Clinical Advisory Committee launched the International Consensus Classification (ICC) of hematologic neoplasms.^[5]^ The ICC classification of specific MN categories was then introduced in detail, such as acute myeloid leukemia (AML),^[6]^ myelodysplastic syndrome (MDS),^[7]^ myeloproliferative neoplasm (MPN),^[8]^ myelodysplasia/myeloproliferative neoplasms (MDS)/MPN),^[9]^ published in Virchows Arch. Therefore, WHO-HAEM4R was updated to 2 different versions at the same time. There are inevitably differences between the 2, and both the classification algorithm and the classification results need to be fully understood.^[10]^ To this end, we used WHO-HAEM5 and ICC to reclassify MN cases previously diagnosed according to WHO-HAEM4R to explore the practical significance of these 2 latest classification systems. We endeavored to carefully examine the contribution of these NGS-detected mutation signatures to both classifications. This is very useful for everyone to understand the different effects of the 2 new classifications in practical applications.

## 2. Materials and methods

### 2.1. Study population

This study included 112 patients with MN diagnosed at the Hematology Department of the Fourth Affiliated Hospital of Zhejiang University School of Medicine from May 2018 to September 2023. The cohort comprised 54 males and 58 females, with a median age of 61 years (range, 18–85 years) (Table 1). All patients underwent a comprehensive diagnostic workup, including complete blood count, white blood cell differential, bone marrow cell morphology, conventional cytogenetics, and mutation analysis using a hematological malignancy NGS panel. Additionally, some patients underwent ancillary studies such as flow cytometry immunophenotyping, fluorescence in situ hybridization, polymerase chain reaction-based fusion gene or mutation analysis, and bone marrow biopsy. The exclusion criteria included patients with incomplete information on bone marrow cell morphology, NGS panel analysis, genetic analysis, and patients who did not meet the WHO-HAEM4R MN diagnostic criteria.^[11]^ The clinical characteristics of patients are presented in Table 1.

**Table 1:** Clinical characteristics of the 112 patients with myeloid neoplasms (WHO-HAEM4R).

	Total	AML	MDS	MPN	MDS/MPN
Patients (n)	112	41	28	36	7
Sex (male/female)	54/58	18/23	14/14	16/20	6/1
Age (median)	60	58	67	51	71
Blood count					
WBC (× 10^9^/L)	7.4 (0.6–333)	8.9 (0.6–333)	3 (0.9–13.2)	6 (4.2–28.3)	10 (3.1–29.4)
Platelets (× 10^9^/L)	99 (1–2209)	70 (10–119)	28 (1–307)	388 (90–2209)	186 (30–703)
Hb (g/L)	101 (43–221)	105 (43–131)	70 (51–136)	170 (123–221)	109 (98–173)
BM blasts (%)	20 (0–96)	56 (12–96)	7.2 (0–19)	1 (0–6)	7.3 (1–16)
PB blasts (%)	1 (0–97)	23 (0–98)	0 (0–8)	1.2 (0–4)	0.7 (0–2)
Karyotype					
Normal	64	17	12	31	4
Altered (complex)	48 (16)	24 (8)	16 (5)	5 (2)	3 (1)
NGS detect mutation	104	38	25	34	7

AML = acute myeloid leukemia, Hb = hemoglobin, MDS = myelodysplastic syndrome, MPN = myeloproliferative neoplasm, WBC = white cell count.

### 2.2. Sequencing and variant annotation

Genomic DNA was extracted from bone marrow or blood samples using the Maxwell RSC Blood DNA Kit (Promega et al., USA). Libraries were prepared using an enrichment-capture gene panel following the manufacturer protocol using 50 ng of DNA. A total of 141 genes associated with hematological diseases were examined, with a mean sequencing depth of 800×. Single nucleotide variants and short fragment indels in protein-coding sequences or exonic hotspots were analyzed using the Ion Reporter™ and Variant Reporter pipelines. Annotation was performed by referencing the dbSNP, 1000 Genomes, Polyphen-2, and COSMIC databases.

## 3. Results

### 3.1. Reclassification using WHO-HAEM5 and ICC

According to the original WHO-HAEM4R criteria, among the 112 MN patients, there were 16 cases of AML with recurrent genetic abnormalities (AML-RGA), 16 cases of AML with myelodysplasia-related changes (AML-MRC), 9 cases of AML not otherwise specified (AML, NOS), 4 cases of MDS with multilineage dysplasia (MDS-MLD), 4 cases of MDS with single lineage dysplasia (MDS-SLD), 17 cases of MDS with excess blasts-1 (MDS-EB1), 3 cases of MDS-EB2, 2 case of chronic myeloid leukemia (CML), 10 cases of polycythemia vera (PV), 13 cases of essential thrombocythemia (ET), 9 cases of primary myelofibrosis (PMF), 1 case of chronic eosinophilic leukemia, not otherwise specified (CEL, NOS), 1 case of myeloproliferative neoplasm-unclassifiable (MPN-U), 5 cases of chronic myelomonocytic leukemia (CMML), 1 case of MDS/MPN with ring sideroblasts and thrombocytosis (MDS/MPN-RS-T), and 1 case of MDS/MPN unclassifiable (MDS/MPN-U).

Using the WHO-HAEM5 classification, there were 18 cases of AML with defining genetic abnormalities (AML-DGA), 17 cases of Acute Myeloid Leukemia, myelodysplasia-related (AML-MR) (Note: Although AML-MR is also included in AML-DGA, as a subtype of AML-DGA, 20% of blast cells are required. It is exceptional and classified separately). 7 cases of AML defined by differentiation (AML-DBD), 1 case of myelodysplastic neoplasm with low blasts and SF3B1 mutation (MDS-*SF3B1*), 7 cases of MDS with low blasts (MDS-LB), 14 cases of MDS with increased blasts-1 (MDS-IB1), 3 cases of MDS with biallelic *TP53* inactivation (MDS-bi*TP53*), 2 cases of MDS-IB2, 2 cases of CML, 10 cases of PV, 13 cases of ET, 9 cases of PMF, 1 case of CEL, 1 case of MPN-NOS, 5 cases of CMML, and 2 cases of MDS/MPN with thrombocytosis and *SF3B1* mutation (MDS/MPN-*SF3B1*-T).

Using ICC, 18 cases of AML-RGA, 8 cases of AML with myelodysplasia-related gene mutations (AML-MRGM), 5 cases of AML with myelodysplasia-related cytogenetic abnormalities (AML-MRCA), 2 cases of AML with *TP53* mutations (AML-*TP53*), 9 cases of AML, NOS, 1 case of MDS-*SF3B1*, 7 cases of MDS, not otherwise specified (MDS, NOS), 14 cases of MDS-EB, 2 cases of MDS/AML with myelodysplasia-related gene mutations (MDS/AML-MRGM), 3 cases of MDS with TP53 mutation (MDS-*TP53*), 2 cases of CML, 10 cases of PV, 13 cases of ET, 9 cases of PMF, 1 case of CEL, NOS, 1 case of MPN-U, 5 cases of CMML, and 2 cases of MDS/MPN-*SF3B1*-T were identified.

**Figure 1.**
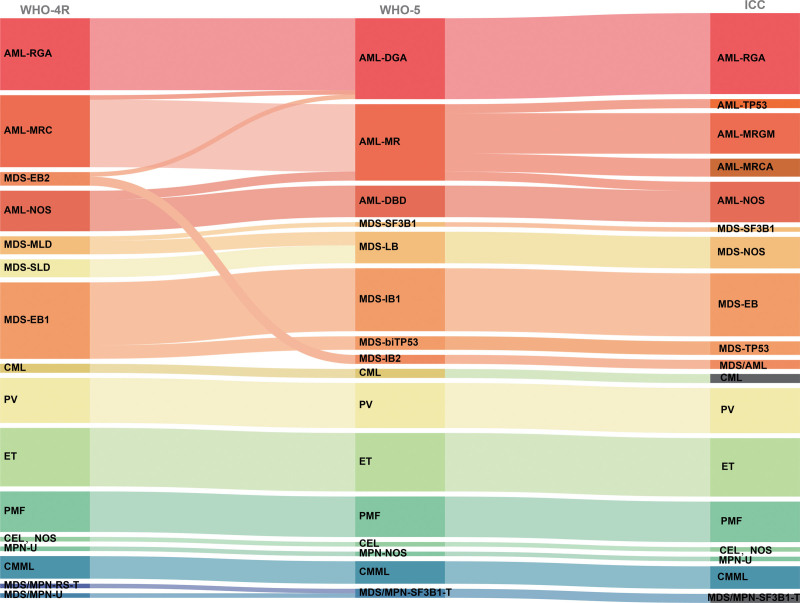
The relationship between WHO-HAEM4R, WHO-HAEM5, and ICC classified MN types. AML-DBD = AML, defined by differentiation, AML-DGA = AML with defining genetic abnormalities, AML-MR = Acute myeloid leukemia, myelodysplasia-related, AML-MRC = AML with myelodysplasia-related changes, AML-MRCA = AML with myelodysplasia‑related cytogenetic abnormalities, AML-MRGM = AML with myelodysplasia‑related gene mutations, AML-RGA = AML with recurrent genetic abnormalities, AML, NOS = AML, not otherwise specified, AML-*TP53* = AML with *TP53* mutations, CML = chronic myeloid leukemia, CMML = chronic myelomonocytic leukemia, ET = essential thrombocythemia, MDS/AML-MRGM = MDS/AML with myelodysplasia-related gene mutations, MDS-bi*TP53* = MDS with biallelic *TP53* inactivation, MDS-EB = MDS with excess blasts, MDS-IB = MDS with increased blasts, MDS-LB = MDS with low blasts, MDS-MLD = MDS with multilineage dysplasia, MDS,NOS = MDS, not otherwise specified, MDS-SLD = MDS with single lineage dysplasia, MDS/MPN,NOS = MDS/MPN, not otherwise specified, MDS/MPN-RS-T = MDS/MPN with ring sideroblasts and thrombocytosis, MDS/MPN-*SF3B1*-T = MDS/MPN with thrombocytosis and *SF3B1* mutation, MDS/MPN-U = MDS/MPN unclassifiable, MDS-*SF3B1*, myelodysplastic neoplasm with low blasts and *SF3B1* mutation, MDS-*TP53* = MDS with *TP53* mutation, PMF = primary myelofibrosis, PV = polycythemia vera.

These 3 classifications are roughly equivalent in classifying these cases, with some differences in terminology. A total of 11 cases had significantly different classifications, including 1 case with only 12% blasts, classified by WHO-HAEM4R as MDS-EB2. In contrast, WHO-HAEM5 and ICC classified it as AML-DGA and AML-RGA, respectively, due to *NPM1* mutations. Two cases were classified as AML-MRC (WHO-HAEM4R) or AML-MR (WHO-HAEM5) due to complex karyotypes; however, in ICC, they were classified as AML-*TP53* because of *TP53* mutation. Two cases of AML-NOS (WHO-HAEM4R) were classified as AML-MR (WHO-HAEM5) and AML-MRC (ICC) because of *ASXL1* and *SRSF2* mutations and *ASXL1* and *U2AF1* mutations, respectively. One case of MDS-MLD was classified as MDS-*SF3B1* in the 2 new classifications because of *SF3B1.* One MDS/MPN-RS-T case and 1 MDS/MPN-U case were reclassified as MDS/MPN-*SF3B1*-T because of *SF3B1* mutation detected (Table 2, Figure 1).

**Table 2:** Defining genetic abnormalities mutations detected by NGS in AML, MDS, and MDS/MPM.

Mutation (number of cases)	Morphological diagnosis	WHO-HAEM5	ICC
*NPM1*(10)	AML	AML-*NPM1*	AML-*NPM1*
*NPM1*(1)	MDS-EB2	AML-*NPM1*	AML-*NPM1*
C*EBPA* bi-mutation(1)	AML	AML-*CEBPA*	AML-*CEBPA*
*ASXL1,BCOR,EZH2,STAG2,SRSF2,U2AF1*(8)	AML	AML,MR	AML-MRGM
*RUNX1*(1)	AML	AML,MR(MDS history)	AML-MRGM
*SF3B1*(1)	MDS-MLD	MDS-*SF3B1*	MDS-*SF3B1*
*SF3B1*(1)	MDS/MPM-U	MDS/MPN-*SF3B1*-T	MDS/MPN-*SF3B1*-T
*SF3B1*(1)	MDS/MPN-RS-T	MDS/MPN-*SF3B1*-T	MDS/MPN-*SF3B1*-T
*TP53*(2)	AML	n/a	AML-*TP53*
*TP53*(3)	MDS	MDS-bi*TP53*	MDS-*TP53*

### 3.2. The genetic mutation landscape of MN

**Figure 2.**
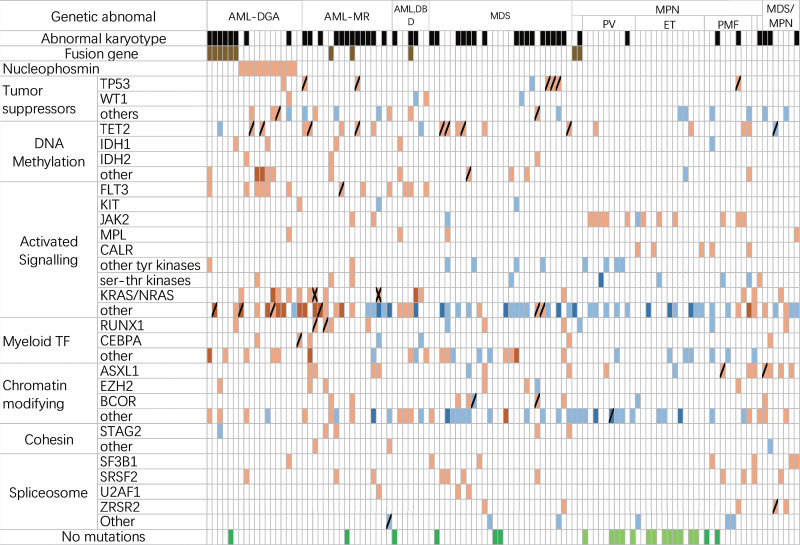
Organization of mutations into categories of related genes (Orange indicates pathogenic mutations, and blue indicates unknown significance. Dark colors indicate mutations in 2 or more genes, slashes indicate double mutations in the same gene, and crosses indicate multiple mutations in the same gene. Dark green indicates a negative result of NGS, and light green indicates “triple-negative” MPN). Other tumor suppressor genes include *RB1, FAT1, SPEN, EGR1, PHF6*; Other DNA Methylation gene include: *SETD2, DNMT3A, DNMT3B, TPMT*; Other tyr kinases gene include: *FGFR3, JAK1, JAK3, PDGFRA, ABL1;* Ser-thr kinases gene include: ATM, BRAF; Other activated signaling gene includ: ABL2, ANKRD26, ATG2, *BCARD11, CBL*,*CCND1, CCND3, CD79B, CSF1R, CSF3R, CSMD1, ECT2L, ETNK1, IL7R, MYB, NF1, NOTCH1, NOTCH2, NOTCH3, NT5C2, PLCG1, PLCG2, PPM1D, PRDM1, PTPN11, RBBP6, RELN, SAMD9, TNFAIP3, TNFRSF14,TRAF3, TYK2*; Other myeloid TF gene include: *CREBBP, CUX1, DDX41, ETV6, GATA1, GATA2, IKZF1, MED12, STAT5A, STAT5B, TAL1, TCF3*; Other chromatin modifying gene include: *ARID1A, ARID5B, ASXL2, BCORL1, DDX41, DIS3, EP300, ID3, KDM6A, KMT2A, KMT2C, KMT2D, PML, SETBP1;* Other cohesin gene include: *RAD21, SMC1, SMC1A, SMC2, SMC3;* Other spliceosome gene include: *PRPF8, SF1, SF3A1*.

In our analysis of 112 MN patients, we detected 104 gene mutations, with only 8 patients showing no mutations. Notably, *TET2, ASXL1, FLT3, FAT1, JAK2*, and *NPM1* emerged as the top 5 most commonly mutated genes in 12 or more patients (Fig. 2). Mutation frequencies varied across MN categories, with rates of 93% (38/41) in AML, 89% (25/28) in MDS, 94% (34/36) in MPN, and 100% (7/7) in MDS/MPN (Table 1).

Figure 2 provides a comprehensive view of the gene mutation landscapes within each patient with MN, considering the WHO-HAEM5 reclassified disease categories.^[4,12]^ Genes were further organized into distinct functional groups, adapted from the relevant literature.^[13]^ When interpreting gene mutation status, it is worth noting that in addition to pathogenic mutations, some mutations of uncertain significance, often with multiple coexisting mutations, are often found in MPN (Table 3, Figure 2).

**Table 3:** NGS-detected mutation genes in “triple-negative” MPN patients.

Diagnosis	NGS-detected mutation genes	Diagnosis	NGS-detected mutation genes
PV	JAK1, KMT2C	ET	KMT2C
PV	KMT2A, KMT2A, KMT2D	ET	RBBP6, CREBBP, PLCG2
PV	TCF3, JAK3, RELN, BCORL1	ET	JAK2*, CD79B, ASXL1
PV	FGFR3, KMT2C	ET	BCORL1, EP300, FAT1
PV	KMT2D	ET	TET2, SAMD9, CUX1, TYK2
ET	ATG2B, NOTCH3	ET	CSF1R
ET	CALR^a^		

ET = essential thrombocythemia, PMF = primary myelofibrosis, PV = polycythemia vera.

a The mutation is not detected by MPN gene panel.

## 4. Discussion

WHO-HAEM5 and ICC are both updates to WHO-HAEM4R. Therefore, 2 new systems are now available for classifying hematolymphoid tumors. This brings inevitable confusion to the clinical diagnosis and subclassification.^[14]^ Comparing the 2 classifications in practice can remind us of situations that require attention. After our reclassification, we found limited differences between the 2 new classifications. The main difference was the addition of disease subtypes, defined by genetic mutations. According to the existing literature, in addition to the differences in this study, there are also some other differences between the 2, such as:

The cutoff for blast cells in some AML subtypes differs between the 2 systems. However, WHO-HAEM5 eliminates the 20% blast requirement for AML by defining genetic abnormalities (except AML with *BCR::ABL1* fusion, AML with *CEBPA* mutation, AML-MR, and AML with other defined genetic alterations).^[4]^ In ICC, blasts must reach 10% or more, except in AML with *BCR::ABL1* fusion, AML-*TP53*, AML-MRGM, AML-MRCA, and AML, NOS.^[5]^ Some AML subtypes require ≥ 10% blast cells in ICC but ≥ 20% in WHO-HAEM5, such as AML with *CEBPA* mutations, AML with *RUNX1T3*(*CBFA2T3*)::*GLIS2*, AML with *KAT6A::CREBBP,* AML with *FUS::ERG*, AML with *MNX1::ETV6*, and AML with *NPM1::MLF1*. In ICC, there are genetic abnormalities such as t(1;3)(p36.3;q21.3)/*PRDM16::RPN1*, t(10;11)(p12.3;q14.2)/*PICALM::MLLT10*, t(16;21)(q24.3;q22.1)/*RUNX1::CBFA2T3*, and ≥ 10% blast cells can diagnose AML subtypes, which are not recognized by the WHO.^[14]^

AML-*TP53* is a unique subtype of ICC that is not recognized as an entity by the WHO-HAEM5. In ICC, as a type of AML with specific genetic abnormalities, its diagnostic priority is between AML-*NPM1* and AML-MRGM. If a patient had *TP53* and *NPM1* mutations simultaneously without other recurrent genetic abnormalities, the patient was diagnosed with AML with *NPM1* and *TP53* mutations. If *TP53* mutation is present along with myelodysplasia-related gene mutations, AML-TP53 should be made.^[5]^

Furthermore, in ICC, pure erythroid leukemia is often associated with *TP53* mutations, and these cases are now classified as AML-*TP53*. However, this classification appears to lack uniqueness, as the clinical features of *TP53*-mutated AML are much broader than the pure erythroid leukemia, which are more homogeneous.^[15]^

In our study, there was no substantial variance in the classification of MPN across the 3 classification systems. Among most triple-negative MPN patients, NGS studies typically identified 2 or more gene mutations concurrently, although occasionally only 1 mutation was discerned (4/17). The mutation profile of triple-negative MPN differs markedly from that of MDS and AML. These mutated genes are not typically found in AML or MDS, and their clinical implications are commonly denoted as having “uncertain significance.” Nevertheless, as a clonal marker, it can serve as a surrogate indicator for *JAK2, CALR*, or *MPL* mutations, which is one of the major criteria for ET and PMF.^[16]^

The primary impact of NGS mutation screening on the diagnosis of MDS/MPN lies in the identification of *SF3B1* mutations, which define genetic abnormalities.

Given the retrospective nature of this study, NGS was selectively performed in patients deemed clinically pertinent, resulting in a relatively modest sample size. The patient distribution exhibited certain biases, such as a higher proportion of triple-negative MPN cases and the absence of myeloid/lymphoid neoplasms with eosinophilia and defining gene rearrangement. However, owing to the inclusion of suspected patients for whom NGS holds the utmost relevance in classification, it achieves maximum efficiency. This underscores the impact of NGS on MN classification to a significant extent.

## 5. Conclusions

Gene mutation analysis of bone marrow samples by the hematological malignancy NGS panel is instrumental for understanding the gene mutation in MN patients and for diagnosing and classifying diseases. Therefore, MN patients is recommended to be tested as much as possible.

## Author contributions

**Conceptualization:** Xiangjun Ye.

**Data curation:** Xiangjun Ye, Zhikang Zheng.

**Formal analysis:** Xiangjun Ye, Zhiping Xu.

**Funding acquisition:** Xiangjun Ye, Jianguo Wu.

**Investigation:** Zhikang Zheng, Zhiping Xu, Jianguo Wu.

**Methodology:** Zhikang Zheng, Jianguo Wu.

**Project administration:** Zhikang Zheng, Yameng Liu.

**Resources:** Zhikang Zheng, Yuwei Wu, Zhihui Zhang, Yameng Liu.

**Software:** Yuwei Wu, Zhihui Zhang.

**Supervision:** Jianguo Wu.

**Validation:** Lei Jiang, Jianguo Wu.

**Visualization:** Xiangjun Ye, Lei Jiang.

**Writing – original draft:** Xiangjun Ye.

**Writing – review & editing:** Xiangjun Ye, Yuwei Wu, Lei Jiang.
